# Supplementary material for: Characterization of arbovirus infections in patients within Haiti by screening discarded malaria rapid diagnostic test cassettes
Source: PLoS Negl Trop Dis. 2026 Mar 20;20(3):e0014089. doi: 10.1371/journal.pntd.0014089 (PMC13004502; doi:10.1371/journal.pntd.0014089)
Supplement: S1 Table — Sample sets in which a sample was determined positive by the pan-flavivirus assay and/or DENV assay are in bold. (DOCX) [file pntd.0014089.s001.docx]

**Supplemental Table 1. Collection months and age of RDTs at time of extraction.** Sample sets in which a sample was determined positive by the pan-flavivirus assay and/or DENV assay are in bold.

| **Year** | **Months (if available)** | **Location (Department)** | **RDT age range (months) before extraction** |
| --- | --- | --- | --- |
| 2021 | June-Sept | Bonne Fin (Sud) | 22-27 |
|  | Dec | CS Collette (Sud) | 23 |
|  | N/A | CityMed Cayes (Sud) | N/A |
|  | N/A | CityMed Petion-Ville (Ouest) | N/A |
|  | May-June | CS FINCA (Sud) | 29-31 |
|  | May-Aug | CS Port-a-Piment (Sud) | 15-24 |
|  | N/A | CS Saint Louis du Sud (Sud) | N/A |
|  | May-Dec | CS Torbeck (Sud) | 25-32 |
|  | N/A | Hopital FCS de P-au-P | N/A |
|  | N/A | Port-Salut (Sud) | N/A |
|  | May-June | Tiburon (Sud) | 16-19 |
| 2021/2022 | May-Dec | Hopital Regional Cayes (Sud) | 23-30 |
| 2022 | Mar-Jun | Hopital de Port Salut (Sud) | 17-18 |
| 2023 | **Aug** | **Dispensarie de Chantal (Sud)** | **15** |
|  | Apr-May | HIC Cayes (Sud) | 3-4 |
|  | **Sept** | **HIC Cayes – Batch 3 (Sud)** | **7** |
|  | **Jun-Aug** | **Hopital de Port Salut (Sud)** | **12-15** |
|  | **Aug** | **Hopital de St. Louis du Sud (Sud)** | **14-15** |
|  | Jul | Hopital de Tiburon (Sud) | 9 |
|  | Apr-Jun | Hopital Les Anglais (Sud) | 4-6 |
|  | **Jun-Jul** | **Hopital Les Anglais – Batch 3 (Sud)** | **12-16** |
|  | **Aug** | **Hopital OFATMA Sud (Sud)** | **15-16** |
|  | Apr-May | Hopital Port-a-Piment – Batch 2 (Sud) | 5-6 |
|  | **Aug** | **Hopital Port-a-Piment – Batch 3 (Sud)** | **12-13** |
|  | Apr | Hopital Port a Piment Public (Sud) | 4-5 |
|  | **Jul** | **Hopital Ste Anne (Sud)** | **12** |
|  | Apr | MSF Port a Piment (Sud) | 8 |
