# Supplementary material for: Characterization of arbovirus infections in patients within Haiti by screening discarded malaria rapid diagnostic test cassettes
Source: PLoS Negl Trop Dis. 2026 Mar 20;20(3):e0014089. doi: 10.1371/journal.pntd.0014089 (PMC13004502; doi:10.1371/journal.pntd.0014089)
Supplement: Attachment — Submitted filename: MSreview_PNTD-25-01951.pdf [file pntd.0014089.s003.pdf]

MS. Review.

Journal:

Manuscript ID: PNTD-D-25-01951

Title: Characterization of arbovirus infections in patients within Haiti by screening discarded malaria rapid diagnostic test cassettes

Authors: Graham A. Matulis et al.

The authors present an innovative approach for arbovirus surveillance in Haiti by repurposing discarded malaria rapid diagnostic tests (RDTs) as a source of nucleic acids for molecular analysis. The study collected 1,884 malaria RDTs from healthcare centers in Haiti's Sud and Ouest Departments between 2021 and 2023. 73 samples were flavivirus-positive or indeterminate, and 68 were confirmed as dengue virus serotype 3 (DENV-3), all from 2023. NGS revealed high genetic similarity (>99.5%) between Haitian DENV-3 strains and those circulating in the Caribbean during 2022–2023. The authors conclude that discarded malaria RDTs can serve as a valuable tool for remote arbovirus surveillance and viral characterization, particularly in resource-limited or insecure settings.

While the manuscript demonstrates an innovative approach, it is important to note that the scenario described—where large numbers of used diagnostic kits are discarded and available for reuse—is not typical in most healthcare settings. Diagnostic kits that have come into contact with clinical specimens pose an infection risk and, under standard protocols, must be sterilized (e.g., by autoclaving) and disposed of as medical waste after use. For this reason, the authors should avoid language suggesting that this method could be generalized or routinely implemented. Instead, the discussion should clearly frame this approach as context-specific and acknowledge the biosafety and regulatory considerations involved.

Furthermore, the manuscript uses RNase P as an indicator of nucleic acid integrity, which is appropriate. However, the interpretation of RNA stability would be more robust if pathogen detection levels were normalized to RNase P Ct values. This would allow the authors to account for sample-to-sample variability and better assess the relationship between RNA degradation and pathogen detection sensitivity. Without such normalization, comparisons across years or seasons are not feasible. Please consider addressing this point.

Therefore, I recommend that this manuscript be considered for publication on PNTD only after major revision, addressing the points outlined below.

Major Points

1. As noted above, the manuscript uses RNase P as an indicator of nucleic acid integrity, which is appropriate. However, the interpretation of RNA stability would be more robust if pathogen detection levels were normalized to RNase P Ct values. This would allow the authors to account for sample-to-sample variability and better assess the relationship between RNA degradation and pathogen detection sensitivity. Without such normalization, comparisons across years or seasons are not feasible.

2. The authors infer the circulation of DENV-3 based on Sanger sequencing of flavivirus-positive samples. However, Sanger sequencing relies on PCR amplification, which tends to favor the most abundant template. Therefore, in cases of mixed infections or low viral load, minority pathogens may not be detected. Please discuss this limitation in the manuscript and consider whether alternative approaches (e.g., deep sequencing or multiplex PCR) could mitigate this bias.

3. The authors tested Alphaviruses and Oropouche virus only in samples that were negative for flaviviruses. However, mixed infections involving flaviviruses and other arboviruses have been reported. Therefore, testing should ideally include all samples regardless of flavivirus PCR results. Please consider expanding the screening strategy or clearly acknowledge this limitation in the discussion.

4. In the Discussion section (Lines 310–314), the authors argue that RNA loss during extraction may have minimal impact because symptomatic patients typically have high viral titers. However, this assumption overlooks scenarios such as mixed infections (e.g., malaria co-infection), where viral copy numbers for flaviviruses or alphaviruses could be significantly reduced. This could lead to false negatives and underestimation of co-circulating pathogens. Please address this limitation in the discussion and consider whether additional normalization or alternative approaches could improve detection accuracy.

Minor Points

1. Materials & Methods: Please revise the Materials and Methods section to ensure that all experimental products include complete manufacturer information, including company name, city, and country, in a consistent format.

2. Line 214: Please clarify the origin of the Plasmodium spp. positive results by PCR. Were these obtained from the current study or based on previously published data? In addition, the manuscript should include a discussion on why 36 samples that were negative by RDT tested positive by PCR. This discrepancy warrants explanation and could provide important insights into diagnostic sensitivity and sample integrity.
