# Supplementary material for: Characterization of arbovirus infections in patients within Haiti by screening discarded malaria rapid diagnostic test cassettes
Source: PLoS Negl Trop Dis. 2026 Mar 20;20(3):e0014089. doi: 10.1371/journal.pntd.0014089 (PMC13004502; doi:10.1371/journal.pntd.0014089)
Supplement: Attachment — Submitted filename: MSreview_PNTD-25-01951_R1_report.pdf [file pntd.0014089.s005.pdf]

Manuscript ID: PNTD-D-25-01951_R1

Title: Characterization of arbovirus infections in patients within Haiti by screening discarded malaria rapid diagnostic test cassettes

Authors: Graham A. Matulis et al.

1. Overall Assessment

The authors have adequately addressed the major and minor comments from Reviewer 1 and the Editor. The revisions improved clarity, safety considerations, and methodological transparency.

2. Key Points Addressed

- Biosafety and context-specific use of discarded RDTs are now clearly explained.
- Methods were expanded with additional details, including reagents, storage conditions, and assay references.
- Limitations regarding mixed infections, RNA degradation, and detection bias were added.
- Ethical approvals and anonymization procedures are now properly documented.

3. Remaining Issues

The normalization of pathogen Ct values to RNase P was not performed; however, the authors' explanation is reasonable and does not affect acceptance.

4. Conclusion

The manuscript has satisfactorily addressed reviewer concerns and is suitable for acceptance.

Date: February 14, 2026
