# Supplementary material for: Characterization of arbovirus infections in patients within Haiti by screening discarded malaria rapid diagnostic test cassettes
Source: PLoS Negl Trop Dis. 2026 Mar 20;20(3):e0014089. doi: 10.1371/journal.pntd.0014089 (PMC13004502; doi:10.1371/journal.pntd.0014089)
Supplement: Attachment — Submitted filename: Response to reviewers (1).docx [file pntd.0014089.s004.docx]

**Editor**

**Although the authors’ study includes some interesting aspects, there are safety concerns regarding the reuse of RDT kits that have already been used for malaria testing or other purposes, such as dengue diagnosis. This point should be clearly stated, as suggested by one of the reviewers.**

> We added language within the abstract, author summary, and conclusion about how this approach is context-specific, requires biosafety precautions to be carried out and was performed in coordination with both local health care centers and government stakeholders. Samples were considered diagnostic with the potential to contain infectious disease agents. Standard infectious disease control measures, such as using a BSC and PPE, were utilized to minimize risk of exposure to potential pathogens. Processing and testing were conducted in an infectious disease institute with BSL-2/3/4 capabilities.

**In addition, many parts of the Methods section are inadequately described. The authors should therefore provide more detailed methodological information and add appropriate references.**

> Additional information was added to the Methods section in response to the reviewers’ requests.

**Reviewer 1**

**While the manuscript demonstrates an innovative approach, it is important to note that the scenario described—where large numbers of used diagnostic kits are discarded and available for reuse—is not typical in most healthcare settings. Diagnostic kits that have come into contact with clinical specimens pose an infection risk and, under standard protocols, must be sterilized (e.g., by autoclaving) and disposed of as medical waste after use. For this reason, the authors should avoid language suggesting that this method could be generalized or routinely implemented. Instead, the discussion should clearly frame this approach as context-specific and acknowledge the biosafety and regulatory considerations involved.**

> We added language within the abstract, author summary, and conclusion about how this approach is context-specific, requires coordination with local health care centers, and requires biosafety precautions to be carried out. Samples were considered diagnostic with the potential to contain infectious disease agents. Samples were stored in a Ziplock bag. Standard infectious disease control measures, such as using a BSC and PPE, were utilized to minimize risk of exposure to potential pathogens. Processing and testing were conducted in an infectious disease institute with BSL-2/3/4 capabilities.

**The objectives of the study are clearly stated, and the manuscript presents a well-defined research question. However, the current version does not include sufficient details regarding the statistical analyses performed to support the conclusions.**

>Besides averages, standard deviation and percent positives, no statistical analyses were performed.

**In addition, there is no explicit description of ethical considerations or regulatory compliance related to the use of clinical specimens. These aspects should be addressed in the revised manuscript to ensure methodological rigor and adherence to ethical standards.**

> Thank you for this comment. All samples collected were clinical discards and were anonymous. This study was reviewed by the George Mason University Institutional Review Board (IRB) and issued a human use determination (OSP #12153A) that the study did not meet the definition of human subject research. Similarly, the USMRIID IRB reviewed the study and determined the study was a human use exempt study (FY24-15). The details of the human use is in the Ethics section (lines 429 – 438). Furthermore, this work is being carried out in close collaboration with the Haitian Ministry of Health (Dr. Jacques Boncy and Dr. Alexandre Existe), who are partnering with our team and approved this study.

**The manuscript uses RNase P as an indicator of nucleic acid integrity, which is appropriate. However, the interpretation of RNA stability would be more robust if pathogen detection levels were normalized to RNase P Ct values. This would allow the authors to account for sample-to-sample variability and better assess the relationship between RNA degradation and pathogen detection sensitivity. Without such normalization, comparisons across years or seasons are not feasible.**

>Unfortunately, given that all DENV detections occurred within a four month period of the same year and batch, this prevents us from doing such comparisons of pathogen detection levels normalized to RNaseP Ct values. This is why we kept our discussion to just averages and standard deviations of the RNaseP Ct values. It would be an interesting study to assess the long-term stability of RNA and DNA pathogens over time to determine decay rates over time. These data could expand the use of this approach but is beyond the funding scope of this current effort.

**The authors infer the circulation of DENV-3 based on Sanger sequencing of flavivirus-positive samples. However, Sanger sequencing relies on PCR amplification, which tends to favor the most abundant template. Therefore, in cases of mixed infections or low viral load, minority pathogens may not be detected. Please discuss this limitation in the manuscript and consider whether alternative approaches (e.g., deep sequencing or multiplex PCR) could mitigate this bias.**

> Discussion of this screening pipeline as an analytical limitation for detecting low abundance pathogens has been added to the limitation section of the paper.

**The authors tested Alphaviruses and Oropouche virus only in samples that were negative for flaviviruses. However, mixed infections involving flaviviruses and other arboviruses have been reported. Therefore, testing should ideally include all samples regardless of flavivirus PCR results. Please consider expanding the screening strategy or clearly acknowledge this limitation in the discussion.**

> Conducting detailed coinfection studies would be very interesting, especially with the malaria-positive samples. However, we are limited in the sample volumes and elution volumes from each sample, requiring us to focus in on a specific pathogen once a positive test result is identified. Language has been added to the limitations section of the discussion to acknowledge this.

**In the Discussion section (Lines 310–314), the authors argue that RNA loss during extraction may have minimal impact because symptomatic patients typically have high viral titers. However, this assumption overlooks scenarios such as mixed infections (e.g., malaria co-infection), where viral copy numbers for flaviviruses or alphaviruses could be significantly reduced. This could lead to false negatives and underestimation of co-circulating pathogens. Please address this limitation in the discussion and consider whether additional normalization or alternative approaches could improve detection accuracy.**

> Thank you for the comment. We added additional information in the limitations section.

**Materials & Methods: Please revise the Materials and Methods section to ensure that all experimental products include complete manufacturer information, including company name, city, and country, in a consistent format.**

> Methods have been updated to include company name, city, and country in a consistent format.

**Line 214: Please clarify the origin of the Plasmodium spp. positive results by PCR. Were these obtained from the current study or based on previously published data? In addition, the manuscript should include a discussion on why 36 samples that were negative by RDT tested positive by PCR. This discrepancy warrants explanation and could provide important insights into diagnostic sensitivity and sample integrity.**

> The Methods and Results sections have been updated. We decided to focus on the non-malaria RDT test results in this study. The *Plasmodium* spp. PCR test results and analyses are beyond of the scope of this manuscript and will be included in a separate manuscript.

**Reviewer 2**

**While this is very well laid out the authors should ensure that the comments provided above are adequately addressed. Please note that on page 7, reference 22 should be reference 23 and many (all?) references after are impacted and should be adjusted as needed.**

> Thank you for catching this. We have updated the references to correct this error.

**How was it decided what samples would be selected? Were all RDTs from the locations indicated used or was it a subset? If the latter, the authors need to describe why the subset is representative of the sites chosen.**

> Samples tested represented a subset of 2,073 total RDTs obtained from Haiti between 2021-2023. The subset not analyzed in this study are samples that are being used for a separate analysis focused only on *Plasmodium* spp. These *Plasmodium* genetic analyses are prioritized and to preserve the limited TNA product obtained from the RDTs, we thus did not test these samples for arboviruses. The samples not included in this study are distributed throughout the different participating health care clinics and did not disproportionately represent one given site. Language is added in the methods section to address this.

**Were the RDT all from the same manufacturer or was there variability? The authors should clarify this and if different kits used how this might impact results. This information should be included in the methods.**

>We have added details to the methods section that all RDTs were either SD Bioline or First Response.

**The storage conditions should be included in the methods. While it is stated in the conclusion that the samples were stored at room temperature it is unclear what this means as this could potentially vary widely. For example, the room may be air conditioned during the day only, or perhaps the room is open air in which room temperature would be expected to be mostly above >27°C in Haiti. It is also unclear how long the samples were stored in Haiti vs Florida.**

> We added the different storage conditions of the RDTs to the methods section.

**Were names or laboratory numbers included on the RDT? The authors should include more information regarding how the RDT were anonymized to the investigators.**

> No identifiable information was provided for the different RDTs. The samples were completely anonymized. More details were given within the methods section about what information was available on the RDT and how those involved with the sample processing/analysis were not given metadata concerning the RDTs.

**It is unclear how the PCR conditions were decided on. If the RNase P amplification was based off of a previous study then then it should be cited. If it was developed in house the authors should provide validation information. For the flavivirus PCR the authors should indicate how the assay was modified and if the modification is expected to lead to different results from the study cited. Are the alphavirus and OROV PCR conditions based off a previous study (if so, cite) or were they developed in house (if so provide validation information).**

> Additional information was added to the Methods section regarding the RNase P assay. The primers and probe are catalog numbers from IDTDNA, and the primers/probe were used according to the TaqPath 1-Step RT-qPCR MasterMix CG kit instructions. The flavivirus real-time PCR assays in the manuscript by Patel and colleagues was adapted for use with the TaqPath 1-Step RT-qPCR MasterMix CG kit. No changes to the primers/probes sequences were made. Additional information was added to the Methods. Assay information was added for the alphavirus spp. and the Oropouche virus real-time PCR assays.

**The authors must elaborate on what an “appropriate PCR curve” is and also indicate what characteristics make a sample indeterminate.**

> We added additional details on what was considered an appropriate PCR curve and details about our designation of an indeterminant sample.

**How were the published sequences chosen to include for phylogenetic analysis and where were the sequences retrieved from?**

> Additional text about the phylogenetic tree analysis were added to the text. Overall, random DENV sequences were selected from NCBI Virus with a maximum of 20 sequences selected per country. Sequences from this study and the random selection of sequences were aligned to build the phylogenetic tree.

**The results are for the most part clearly presented. However, Figure 2 could benefit from a more logical layout in which it is more obvious that the blown up phylogenetic tree is coming from the larger phylogenetic tree. It would also be beneficial to label the sequences shown in this study as “Haiti”. It is not clear or some clades if the country designation is indicating a tip or a clade (e.g., China, India, French Guiana).**

> Thank you for the comment on the figure presentation. We updated the figure to show the tree in more detail. Specifically, we highlighted the area of the tree that was enlarged in green, and we indicated in the enlarged part of the tree the Haiti samples.

**There were 73 flavivirus positive/indeterminate samples and 68 identified as DENV-3. Were the 5 samples not identified as DENV-3 indeterminate? The authors should provide a statement regarding these 5 samples (and the interpretation of an indeterminate should be described in the methods as described above).**

> These 5 samples were not found to be DENV-3 indeterminate. A sentence added to the results section detailing that Sanger sequencing was performed to try to identify the flavivirus in these samples.

**How were the subset of samples decided to run for alphavirus PCR, OROV PCR, and DENV WGS? Could there be bias?**

>Alphavirus and OROV testing were conducted on flavivirus-negative samples that were randomly selected using a random number generator for the sample IDs. The amount of samples tested for each pathogen was relative to the number of samples from each location. We focused this randomized testing strategy on the samples from batch 3 (2023) since we believed there was an impact on viral recovery from RDT age and samples from batch 3 would have been the most promptly processed samples. Language has been added to the methods section to clarify this. The subset of samples for DENV WGS was selected to reflect all health centers from which there were DENV-positive samples with lower (≤36) Ct values to increase the chances of obtaining high quality sequences. Language has been added to the methods section to reflect this schema.

**First sentence. The authors should explicitly state that the positivity rate is for symptomatic people testing for malaria to avoid any confusion with interpretation of positivity rate.**

> We added details throughout the manuscript to specify that these samples were specifically from febrile patients and not the general population.

**ZIKV typically has a much lower viremia than CHIKV and DENV. The authors should mention this in the discussion as it is likely that ZIKV could be missed using the RDT approach as it can even be difficult to identify in an infected person (low viremia and short duration of viremia)**

> We added such details to our limitations section, using what is known about ZIKV viremia levels and temporal duration to illustrate the potential viral TNA recovery weakness.
